# Comparison of CEA and IgG serum levels in oral lichenoid lesions before and after treatment with topical corticosteroids

**DOI:** 10.34172/joddd.2022.022

**Published:** 2022-10-15

**Authors:** Maryam Hosseinpour Sarmadi, Ali Taghavi Zonouz, Aila Bahramian, Amir Ghorbanihaghjo, Farshad Javadzadeh

**Affiliations:** ^1^Department of Oral Medicine, Faculty of Dentistry, Tabriz University of Medical Sciences, Tabriz, Iran; ^2^Biochemistry & Clinical Laboratories Department, Biomedical Research Institute, Tabriz University of Medical Sciences, Tabriz, Iran

**Keywords:** Carcinoembryonic antigen, IgG, Local steroids, Oral lichenoid lesions, Visual analog scale

## Abstract

**Background.** Lichen planus is considered a potentially malignant condition with an unknown etiology. This study aimed to determine the carcinoembryonic antigen (CEA) and IgG serum levels in different oral lichenoid lesions before and after treatment with local corticosteroids.

**Methods.** Two groups of 23 individuals, including oral ulcerative lichenoid lesions patients and healthy ones, were evaluated. Toluidine blue staining and biopsy examinations were carried out while visual analog scale (VAS) was used to evaluate symptoms. By applying corticosteroids, CEA and IgG serum levels were determined before and three weeks after intervention and at the end of the study (9 weeks) with ELISA and turbidimetry methods, respectively.

**Results.** Before the intervention, there was no significant difference in CEA serum levels between the control and case groups (*P*=0.19). Moreover, the CEA serum levels indicated no significant difference before and after treatment in the case group (*P*=0.30). While IgG serum level was significantly higher before the intervention (*P*=0.01), it decreased significantly in the case group after treatment (*P*=0.02). In addition, pain intensity reduced significantly in the case group (*P*=0.05). According to statistics, 8.2% out of 21.7% of patients with positive staining results exhibited dysplasia signs.

**Conclusion.** However, neither CEA nor IgG serum levels were different in patients diagnosed with or without dysplasia and positive or negative staining results (*P*>0.05). IgG serum levels and pain severity effectively decreased in the oral ulcerative lichenoid lesions patients treated with local corticosteroids. Therefore, this treatment can be considered an effective and low-complication treatment modality for lichenoid lesions.

## Introduction

 Oral lichenoid lesions have different etiologic factors but exhibit similar clinical and histologic views. These lesions include lichen planus, contact lichenoid reaction, lichenoid drug reactions, and GvHD (graft versus host disease) lichenoid reaction.^1^ The etiology of lichen planus is still unknown. Many studies have reported a high prevalence of this condition, with 0.5%–2.5% of the population being affected. The condition is more prevalent in middle-aged individuals and women.^2^ Despite contradictory results, oral lichenoid lesions have been reported to be premalignant lesions, with a potential to progress to squamous cell carcinoma (SCC). Different studies have reported different rates of malignant changes, with a range of 1.4–3.8%.^3^ An early diagnosis of premalignant and malignant lesions of the oral mucosa has a significant role in improving patients’ prognosis and life span.^4^ Different serum and salivary biomarkers have been reported for the early diagnosis of dysplasia in premalignant lesions and the risk of malignancy.^5^ One of the most critical markers is carcinoembryonic antigen (CEA) glycoprotein with a role in cell adhesion, which has been evaluated in many malignancies. It has been demonstrated that an increased serum level of CEA, albeit nonspecifically, is associated with the progression of malignant conditions, which might be a factor in the initial diagnosis, disease recurrence, and the control of treatments for malignancies. He et al^6^and Honarmand et al^7^ showed significantly higher CEA levels in oral SCC. In addition, a study by Zheng et al^8^ on the serum and salivary levels of CEA in premalignant conditions (including oral lichen planes, leukoplakia, erythema, and SCC) showed increased serum and salivary levels of CEA in malignancies. In the present study, the CEA tumor marker was evaluated in different lichenoid reactions with symptomatic malignant potential. In addition, the results were compared with the results of biopsies and toluidine blue staining.^9^ Since no definitive treatment is available for lichen planus, and treatment with local steroids is the first line of symptomatic treatment,^1^ in the present study, CEA and IgG serum levels and visual analog scale (VAS) scores were evaluated before and after treatment with local steroids.

## Methods

 In the present descriptive-analytical study, 25 patients with oral erosive lichenoid lesions, referring to the Department of Oral and Maxillofacial Medicine, Tabriz Faculty of Dentistry, were included as the case group patients based on similar previous studies^8^ and using the samples size estimation formula. Finally, 23 patients completed the study. In addition, 23 healthy individuals were included as the controls. All the procedures followed were in accordance with the ethical standards of the committee responsible for human experimentation (institutional and national) and with the Helsinki Declaration of 1975, as revised in 2000. Informed consent was obtained from all the patients for being included in the study. The lesions were confirmed based on clinical symptoms and signs by an oral and maxillofacial medicine specialist, followed by toluidine blue staining, biopsy, and histopathological evaluations. Applying toluidine blue entails four steps: The first step is rinsing the oral cavity with water for 20 seconds to remove debris; the second step is rinsing with 1% acetic acid for 20 seconds; the third step is the application of toluidine blue solution (1% W/W) for 20 seconds; the last step is to rinse with 1% acetic acid for 20 seconds to mechanically eliminate retained stains.^10^ Finally, the lesions were diagnosed based on histopathological criteria and evaluation of dysplasia from mild to severe.^11^ VAS was used to determine the patients’ pain severity before and after treatment. To this end, a 10-cm line was drawn on paper, and each patient was asked to mark their pain severity without any stimulus. The severity of pain was graded from the left at zero (no pain) to 10 on the right (severe pain) and measured with a ruler to record the numeric value as a VAS score.^9^ In the last stage, venous blood samples were collected from the patients in the fasting state. IgG serum levels were determined using the turbidimetric method, and CEA serum levels were determined using competitive ELISA before treatment, three weeks after treatment, and nine weeks after treatment. Statistical significance was set at *P* < 0.05.

###  Inclusion criteria

 Patients with different lichenoid reactions with symptomatic malignant potential, including erosive lichen planus, contact erosive lichenoid reaction, and erosive lichenoid drug reactions based on clinical and histopathological criteria.^12^

###  Exclusion criteria

 Patients with medical conditions that might affect CEA serum levels, including other malignant and premalignant conditions, use of tobacco,^6,7^ and conditions affecting IgG, including congenital and acquired defects of the immune system such as AIDS, chemotherapy, addiction to injection drugs, etc., and the individuals unable to tolerate the complications of biopsy procedures and infection control procedures, individuals undergoing treatment for lichen planus during the past two months.

 The controls were selected from healthy individuals without lichen planus and other systemic diseases, referred for routine dental procedures. The exclusion criteria for the controls were similar to the case group. The case and control groups were matched in terms of age and gender.

 Treatment in the case group: CEA and IgG serum levels were determined before initiating treatment (at most five days). Then the patients underwent routine treatment that consisted of using local steroids in the form of a combination mouth rinse, including aluminum-magnesium syrup and betamethasone vials (n = 15) for three weeks, followed by tapering for the next six weeks. Three and nine weeks after the initiation of treatment, blood samples were collected again to determine the CEA and IgG serum levels, and the results were compared with the baseline CEA and IgG serum levels.

## Results

**Table 1** presents a comparison of the subjects in the case and control groups in terms of the patients’ numbers in both genders, the patients’ age, and the lichen planus type in the cases. As shown in the table, there were no significant differences in gender and the number of patients between the two groups.

**Table 1:** The particulars of the patients and control groups

	**Group**		***P*** ** value**
	**Case**	**Control**	
Female, No. (%)	13 (56.5)	12 (52.2)	0.50*
Male,	10 (43.5)	11 (47.8)	
Age, Mean (range)	59 (29-79)	53 (29-77)	0.18**

*Chi-square (*P* < 0.05). **Mann-Whitney U (*P* < 0.05).

 The mean of IgG levels in the cases before treatment (15.69) was significantly higher than that in the healthy subjects (9.92) (*P* = 0.01). Regarding CEA, the means in the cases and controls were 2.49 and 1.7, respectively, and the difference was not significant (*P* = 0.19) (Table 2).

**Table 2:** A comparison of CEA and IgG serum levels in patients with lichen planus and the controls before treatment

	**Group**	**Mean**	**(Min-Max)**	***P*** ** value**
IgG (before) (g/L)	Patient	15.69	(9.12‒40.65)	0.01^*^
	Control	9.92	(7.49‒18.26)	
CEA (before) (ng/mL)	Patient	2.49	(0.5‒6)	0.19^*^
	Control	1.7	(0.4‒3.91)	

*Mann-Whitney U (*P* < 0.05).

 In the case group, IgG serum levels changed significantly during treatment (*P* = 0.02) (Table 3). Also, in this group, CEA serum levels changed during treatment, but these changes were not significant (*P* = 0.02) (Table 4).

**Table 3:** Comparison IgG serum levels in patients with lichen planus during treatment

	**Min**	**Max**	**Mean**	***P*** ** value**
IgG (before) (g/L)	9.12	40.65	15.69	0.02^*^
IgG2 (g/L)	8.06	34.90	13.3	
IgG3 (g/L)	6.05	29.94	14.27	

*Friedman test (*P* < 0.05).

**Table 4:** The trend of changes in CEA serum levels during treatment

	**Min**	**Max**	**Mean**	***P*** ** value**
CEA (before) (ng/mL**)**	0.50	6.00	2.49	0.3^*^
CEA2 (ng/mL)	0.57	34.00	2.14	
CEA3 (ng/mL)	0.80	5.33	2.12	

*Friedman test (*P* < 0.05).

 A comparison of the treatment outcomes with local steroids in lichen planus patients concerning IgG serum levels and pain severity based on VAS showed the potential of this treatment modality to decrease serum levels of IgG up to the normal level and decrease pain severity significantly (Tables 5 and 6).

**Table 5:** IgG serum levels (control vs. case group) after treatment

**Parameter**	**Control group**	**Case group (after treatments)**	***P *** **value**
IgG (g/L)	9.9 (7.49‒18.26)	14.27 (6.05‒29.94)	0.05^*^

*Mann-Whitney U (*P* < 0.05).

**Table 6:** Pain severity based on VAS in case and control groups

**Parameter**	**Case group (before treatment)**	**Case group (after treatments)**	***P*** ** value**
VAS	5.5 (1‒10)	1 (0‒5.5)	0.01^*^

*Mann-Whitney U (*P* < 0.05).

**Figure 1:**
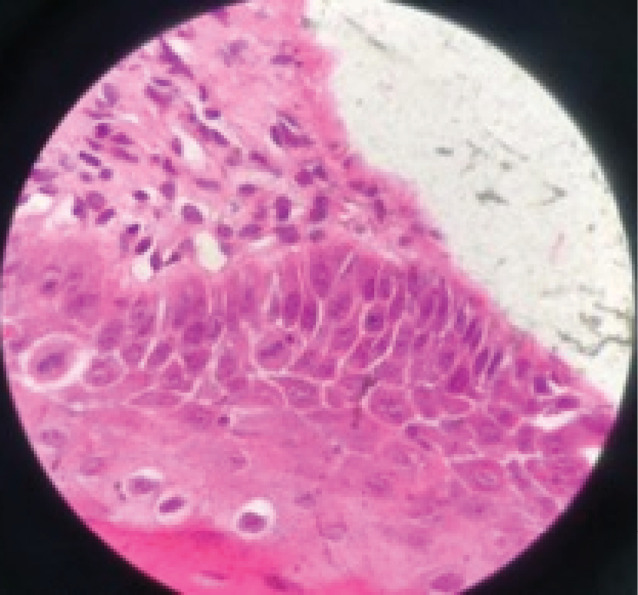
Histopathologic features of the oral lichen planus biopsies with mild dysplastic changes (H&E stain).

 Around 78% of patients were negative for toluidine blue staining with no dysplasia, and 22% were positive for toluidine blue staining [9% mild dysplasia (Figure 1) and 13% without dysplasia]. In two patients with mild dysplasia, the mean IgG serum level before treatment was 18.5 g/L (15.08‒21.92), which was not significantly different (*P* = 0.74) from patients without dysplasia [15.69 g/L (9.12‒40.65)]. In addition, CEA serum levels before treatment in patients with and without dysplasia were 1.74 (0.78‒2.70) and 2.49 (0.50‒6.00) ng/mL, respectively, with no significant differences (*P* = 0.48). The mean serum IgG levels in patients with dysplasia before and after treatment were 18.5 (15.08‒21.92) and 20.60 (11.26‒29.94) g/L, respectively, with no significant differences (*P* = 0.66). However, there was a significant difference in IgG serum levels in patients without dysplasia before treatment [15.69 (9.12‒40.65) g/L] and after treatment [14.27 (6.05‒28.14) g/L] (*P* = 0.01). The CEA serum levels in patients with dysplasia before and after treatment were 1.74 (0.78‒2.70) and 2.03 (1.96‒2.10) ng/mL, respectively (*P* = 0.66). The mean serum levels of this marker in patients without dysplasia before and after treatment were 2.49 (0.50‒6) and 2.20 (0.8‒5.33) ng/mL, respectively, with no significant differences (*P* = 0.95).

## Discussion

 Although tumor marker levels change in cancers, increased levels of tumor markers are not sufficient to diagnose cancers; a combination of these markers with special diagnostic procedures is considered a diagnostic protocol. However, tumor markers can be used for screening and early diagnosis of cancers, determining the disease progression, monitoring treatment, and detecting recurrence.^13^ The present study showed no significant differences in CEA serum levels between the case and control groups before treatment. In addition, after routine local steroid treatment, there were no significant changes in CEA serum levels. Several studies have evaluated the CEA marker levels in oral SCC and premalignant lesions of the oral cavity. Grimm et al^14^ reported no significant changes in anti-CEA during the clinical course of cancers. Li et al^15^ reported significantly higher CEA salivary levels and in the local cells removed in layers from the oral SCC tumors in patients compared to non-cancer patients, with no significant difference in its serum levels between the two groups. They reported that it was possible to evaluate the salivary CEA levels as a reliable indicator for the early diagnosis of oral malignant lesions. Although most previous studies are consistent with the present study, Zheng et al^8^ reported different results, indicating increased salivary and serum levels of CEA in malignancies, including the oral lichen planus, leukoplakia, and oral SCC. In addition, the study above showed a relationship between salivary CEA levels and the clinical stages and lymph node metastasis; however, the serum levels of CEA were related only with lymph node metastasis. A possible reason for the differences in the results might be the target groups; in the present study, only the lichenoid lesions were evaluated, and in the study by Zheng et al,^8^ leukoplakia and other malignancies were evaluated. Elabany et al^16^ evaluated IgG, IgM, and IgA immunoglobulins and CEA in OLP with or without epithelial dysplasia to predict premalignant potential. The results showed a relationship between immunoglobulins and CEA in all the OLP cases with different degrees of epithelial dysplasia. However, there were no significant differences in CEA and IgG serum levels between patients with and without dysplasia in the present study. Such a discrepancy might be attributed to the low frequency of patients with dysplasia in the present study, in which all the dysplasia cases were mild, and possibly, this degree of dysplasia in these patients did not help achieve a significant increase in CEA in the case group. The present study showed significantly higher serum IgG levels in the case group before treatment than in the control group. In addition, in the case group, the treatment of lichen planus with local steroids decreased IgG levels. Therefore, serum IgG levels after treatment were significantly lower than those before treatment. Sistig et al^17^ reported increased mean IgG and IgA serum levels in patients with lichen planus compared to healthy subjects. In addition, Gandolfo et al^18^ reported increased mean serum IgG levels in patients with erosive-atrophic lichen plans than patients with reticular lichen planus, confirming the role of the humoral immune system in the pathogenesis of lichen planus. Mehdipour et al^19^ reported higher IgG levels in patients with ulcerative oral lichen planus than the non-ulceration cases and the control group. Despite the consistency in most previous studies and the present study, Khan et al^20^ showed lower serum levels of all the immunoglobulins in patients with lichen planus than the control subjects. In a study by Albanidou-Farmaki et al,^21^ IgG and IgM serum levels in patients with lichen planus were not significantly different from the control group. However, the serum IgA levels in these patients were higher than in the controls.

 In the present study, three patients were positive for toluidine blue stating with no dysplasia, indicating false-positive toluidine blue staining. Two patients had real positive results for staining, and 18 patients with negative toluidine blue staining results did not exhibit dysplasia, indicating that toluidine blue staining does not have false-negative results. According to a study by Kim et al,^22^ inflammatory and ulcerative lesions (irrespective of their malignant nature) tend to retain stains due to cell activity and mechanical retention, leading to false-positive results, with higher sensitivity and lower specificity for toluidine blue staining. In explaining the discrepancies in the results between the present study and the studies above, it is necessary to note that lichen planus has an unknown etiology despite its high prevalence. It appears that ethnic, geographic, and environmental factors are involved in its etiology. The study population evaluated in the present study differed in the three factors mentioned above (ethnicity, geography, and environment) from the populations in other studies mentioned above.

 In addition, the confounding factors in the present study, including age, gender, lichenoid lesion types (ulcerative and non-ulcerative) in the present study were different from those in the studies mentioned above.

## Conclusion

 In the present study, local corticosteroids were used to treat patients as the first line of treatment for oral lichen planus. The serum IgG levels in lichen planus patients were higher than the healthy individuals, which decreased to almost normal levels after treatment. However, the treatment did not affect the CEA serum levels. Therefore, it can be concluded that this treatment modality decreased the patients’ symptoms based on VAS and decreased inflammatory processes (decreased IgG levels) but did not affect the precancerous condition of the lesions. Although topical corticosteroids have been proposed as a relatively effective treatment with few side effects and even considered by some researchers as the first line of treatment, no research has investigated its effect on CEA and IgG. Therefore, the present study emphasizes again that it has little harm. Finally, we suggest that if a longitudinal study of 10‒15 years can be performed with more patients, more facts about the disease will be revealed. We also suggest that the response to treatment can be compared to topical and systemic corticosteroids in these patients.

## Acknowledgments

 None.

## Authors’ Contributions

 MHS, ATZ, and AB contributed to the study concept and design. MHS, ATZ, and AG contributed to material preparation, data collection, and data analysis. AB and FJ contributed to acquisition and critically revised the manuscript. All the authors commented on previous versions of the manuscript. Finally, all the authors read and approved the final manuscript.

## Funding

 This work was funded by the Drug Applied Research Centre of Tabriz University of Medical Sciences, Tabriz, Iran.

## Ethics Approval

 The Ethics Committee of Tabriz University of Medical Sciences approved the protocol of the study under the code IR.TBZMED.REC.1399.550.

## Competing Interests

 The authors disclose no conflicts of interest.
